# Update on the Mechanisms of Tubular Cell Injury in Diabetic Kidney Disease

**DOI:** 10.3389/fmed.2021.661076

**Published:** 2021-03-30

**Authors:** Jingsheng Chang, Jiayi Yan, Xueling Li, Ni Liu, Rong Zheng, Yifei Zhong

**Affiliations:** Department of Nephrology, Longhua Hospital, Shanghai University of Traditional Chinese Medicine, Shanghai, China

**Keywords:** tubulointerstitial fibrosis, diabetic kidney disease, proximal tubular cell, proximal tubular, pathogenesis

## Abstract

Increasing evidence supports a role of proximal tubular (PT) injury in the progression of diabetic kidney disease (DKD), in patients with or without proteinuria. Research on the mechanisms of the PT injury in DKD could help us to identify potential new biomarkers and drug targets for DKD. A high glucose transport state and mismatched local hypoxia in the PT of diabetes patients may be the initiating factors causing PT injury. Other mechanism such as mitochondrial dysfunction, reactive oxygen species (ROS) overproduction, ER stress, and deficiency of autophagy interact with each other leading to more PT injury by forming a vicious circle. PT injury eventually leads to the development of tubulointerstitial inflammation and fibrosis in DKD. Many downstream signaling pathways have been demonstrated to mediate these diseased processes. This review focuses mostly on the novel mechanisms of proximal renal tubular injury in DKD and we believe such review could help us to better understand the pathogenesis of DKD and identify potential new therapies for this disease.

## Introduction

Diabetic kidney disease (DKD) is a progressive microvascular complication of diabetes mellitus. Within the kidney, the glomeruli, tubules, vessels, and interstitium are disrupted, resulting in impaired renal functions and eventually end-stage renal disease (ESRD). Epidemiological studies have shown that the current global burden of diabetes affects more than 425 million people. Without intervention, the number of individuals with diabetes worldwide will rise to an estimated 629 million in 2045 (1). Given the high prevalence of diabetes, accordingly, the incidence of DKD is rising rapidly with ~30–40% of diabetic patients develop DKD and a third of these patients progress to ESRD, which brings tremendous impacts on the socio-economics (2, 3).

Based on the distinct pathological glomerular changes (4), DKD has previously been regarded as a glomerular disease, and the injury to the renal tubules considered as secondary to glomerular lesions. In the natural history of DKD, the development of persistent microalbuminuria (presence of albumin in the urine) progresses to overt proteinuria, followed by a gradual decline in the glomerular filtration rate (GFR) and eventually renal failure (5). Although albuminuria represents an independent risk factor for DKD, about 20% of patients with non-albuminuric DKD progress to advanced ESRD within 10 years (6). The in-depth understanding of the disease has enabled the identification of some patients with decreased renal function before the presence of microalbuminuria according to creatinine-based estimated glomerular filtration rate (eGFR) (7–10), and these are the patients that tend to progress more rapidly. When compared with patients with proteinuria, these patients tend to have more severe tubulointerstitial fibrosis and tubular atrophy, suggesting that renal tubular injury plays a key role in the progression of DKD in the absence of proteinuria. In general, renal tubular injury is closely correlated with the decline of eGFR in chronic kidney disease (CKD) patients. Recent evidence has suggested that the proximal tubular (PT) injury develops in the early stage of DKD and promotes DKD progression (11). Therefore, this review focuses on the mechanisms of PT injury in DKD.

## Hypoxia

The kidney is an oxygen-intensive organ that receives 20% of the cardiac ejection fraction. The activity of renal tubular transport is accountable for major oxygen consumption in kidney metabolism. The process of renal oxygenation consists of a fine and balanced physiological process, which includes oxygen supply determined by renal blood flow as well as arterial oxygen content and oxygen consumption governed by renal tubular reabsorption. The oxygen in the renal cortex is mainly utilized for glomerular filtration and solute reabsorption. Majority of oxygen supply goes to the renal cortex with a low supply to the renal medulla. This ensures an effective countercurrent multiplication system while the oxygen supply of the medulla is extremely limited, albeit slightly higher than its oxygen utilization (12). Therefore, an imbalance between the oxygen supply and oxygen demand in the medulla will result in hypoxic damages to the medulla tubules (13), whereby the renal tubules in the medulla are the most vulnerable to renal ischemic injury (14).

The development of hypoxia depends on three factors: increased oxygen consumption, oxygen utilization disorder, and reduced oxygen supply, which often co-exists simultaneously and interacts with each other to form a vicious circle (15). Studies have shown that 60% of the overall energy consumption of kidneys is devoted to sodium reabsorption with the PT responsible for almost two-thirds through basal Na^+^/K^+^ ATPase activity primarily and quantified as ouabain-sensitive O_2_ consumption (16). The glucose in the tubule fluid is delivered into the cell by secondary active transport mostly via the sodium-dependent glucose transporters 2 (SGLT-2) in the apical membrane of the proximal tubular epithelial cell (PTEC). Although this is not an energy-dependent process, the sustainability of this activity demands a persistent electrochemical gradient of Na+ produced by Na^+^/K^+^ ATPase activity. Therefore, excessive glucose reabsorption in PT will invariably lead to increased oxygen consumption in type 2 diabetes (17). Moreover, the diabetic kidney is constantly in a state of high oxygen consumption due to hyperfiltration and increase tubular reabsorption, which increases further the severity of renal tubular hypoxia. This situation is then exacerbated by subsequent mitochondria dysfunction (15). Besides, the most classic complication of diabetes is systemic microangiopathy which is characterized by basement membrane thickening with hyaline deposition. This vascular injury will lead to decreased blood supply and oxygen supply in the kidney (18, 19). Furthermore, gluconeogenesis is a major source of oxygen and energy consumption in the kidney, accountable for 25% of the energy required for sodium reabsorption (20). For diabetic kidneys, the degree of gluconeogenesis in the kidney is increased significantly (14, 21).

In studies using diabetic animal models, outer medullary hypoxia has been demonstrated using blood oxygen level-dependent (BOLD) MRI. Also, both cortical and medullary hypoxia has been reported in the diabetic animal models as well as humans with DKD (22–25). With increased oxygen utilization, hypoxia inducible factor (HIF)-1 α has been implicated in the correlation of hypoxic and tubulointerstitial fibrosis (26–30). On the other hand, SGLT-2 inhibitors have been shown to poses a renal protective effect on diabetes patients by inhibiting glucose reabsorption and its associated high oxygen consumption (21, 31), in addition to targeting HIF-1 α protein to inhibit mitochondria oxygen consumption (32, 33). Furthermore, these factors inhibit and internalize megalin O-GlcNAcylation to reduce the reabsorption of plasma proteins (e.g., albumin and neutrophil gelatinase-associated lipoprotein) in PT, which is renal protective (34).

## Mitochondrial Dysfunction

PTEC demands substantial energy to maintain a normal function (35), whereby 65% of the electrolytes and 100% of the glucose and amino acids filtered by the glomeruli are reabsorbed by the PT. PTEC is rich in mitochondria which is an important organelle performing oxidative metabolism in eukaryotic cells mainly through the β-oxidation of fatty acids to produce adenosine triphosphate (ATP) (35). Mitochondria is also a place for aerobic respiration and energy supply of cells, which produces 95% of the energy needed in cellular activities through oxidative phosphorylation and therefore is regarded as the power plant of a cell. Mitochondria is the center of ATP production and its dysfunction leads to apoptosis.

Mitochondrial homeostasis is strictly essential for an optimally functioning kidney, given that the kidney is an organ that demands high energy consumption (36). In diabetes, the epithelial cells of the S1 segment of the PT require a large amount of ATP as an energy source to reabsorb excess glucose. However, ATP production brings superoxide (${\text{O}}_{2}^{-}$) production concurrently, which can be converted into excessive reactive oxygen species (ROS), leading to mitochondrial damage and disorders in ATP production (36). Indeed, a reduction in the ATP pool represents the initial event of PTEC damage, with the degree of ATP reduction correlates with the severity of the damage (37). Studies have demonstrated that the production level of ROS may exceed the capacity of the local antioxidants, which is the biomarker of renal mitochondrial dysfunction in diabetes (38–41). This is further supported by the changes of bioenergetics and kinetics of mitochondria that may precede the development of DKD (38).

In addition to the driving force of cells, mitochondria have also been regarded as the judge and executor of programmed cell death (42, 43). In mitochondrial homeostasis, a balance in the mitochondrial biogenesis, including fusion and mitophagy, is required (35). Both Fission and fusion complement each other to maintain the mitochondrial morphology under different metabolic conditions, while mitophagy removes damaged mitochondria from the network (35). Mitochondrial swelling is considered an indicator of mitochondria dysfunction (44), which can be confirmed by electron microscopy (45, 46). Uncontrolled mitochondria dysfunction eventually leads to the activation of the intrinsic cell death pathway and cell death (47, 48). Cell death may present in various forms, including apoptosis, autophagic cell death, pyroptosis (49).

In recent years, increasing research studies have been performed on the role of oxidative stress in cell death, given its integral role in tubule injury in DKD. Studies have shown that AOPPs (50, 51) induces oxidative stress and DKD mitochondria dysfunction through CD36/β-Catenin and PKC pathways, leading to tubulointerstitial fibrosis. On the contrary, in animal studies using DKD mice, PGC-1 α (52) ameliorates renal fibrosis via an antioxidant mechanism. Antioxidants (tempol and ramipril) inhibit NADPH upregulation by negatively regulating the endoplasmic reticulum stress (ERS) and inflammation to improve renal damage in DKD (53). Oxidative stress and endoplasmic reticulum stress positively regulate by each other, forming a vicious cycle (54). Sirt3-CD38 has also been shown to play a role in diabetic renal tubule damage by regulation of mitochondrial oxidative stress (55, 56).

Given that the mitochondria may be a target for therapeutic intervention, the mechanisms of some potential drugs have been explored. SS31, a novel antioxidative peptide that targets mitochondria, has been specially designed to concentrate in the inner mitochondrial membrane (57), which reduces renal tubulointerstitial damage in diabetic mice by decreasing mitochondrial fragments and restoring mitochondrial morphology through the inhibition of Drp1 expression and upregulation of Mfn1 expression in renal tubular epithelial cells. Also, the role of SS31 has been associated with CD36 (58). Besides, Na2S4, a polysulfide donor that directly sulfhydrates SIRT1, reduces high glucose-induced oxidative stress, cell apoptosis, inflammatory response in renal tubular epithelial cells, and the progression of epithelial-to-mesenchymal transition (EMT) (59). Also, Carnosine has been shown to significantly decrease the production of ROS, alleviate oxidative stress, and inhibit apoptosis through mitochondrial pathway *in vitro* (60) and *in vivo* (61). This may be a promising drug for the treatment of DKD. All these studies shed light on the new potential therapeutic agents in the prevention of renal tubulointerstitial damage through regulation of mitochondrial function and ROS production.

## Innate Immunity

A persistently high glucose can cause abnormal activation of mitochondrial endoplasmic reticulum stress and intracellular signal transduction pathways, leading to cell stress and cellular dysfunction. The abnormal activation following each stress response promotes further activation of downstream inflammatory factors, the release of damps, and induction of innate immune response. The innate immune response induces a continuous process of chronic inflammatory reaction in the kidney, leading to substantial mesangial hyperplasia and renal interstitial fibrosis, which lays the foundation for the occurrence and development of DKD (62). Compared with adaptive immunity, the mechanism of an innate immune response plays an integral role in the occurrence of diabetic kidney injury (63, 64), which is composed of pattern recognition receptors that recognize pathogenic and endogenous ligands. The bindings of ligands trigger several complex inflammatory cascade reactions, including Toll-like receptor (TLR) signaling, nucleotide-binding domain and leucine-rich repeat containing receptors (NLRs), the kallikrein–kinin system (KKS), protease-activated receptor (PAR) signaling, and the complement cascade, resulting in further renal fibrosis and other renal damages (65). In particular, the complement cascade plays a key role in innate immunity that is responsible for the pathogenesis of several immune-mediated inflammatory diseases (66). A study has shown that the novel aptamer (NOX- D21) improves renal function and reduces tubulointerstitial fibrosis by inhibiting the expression of C5a in db/db mice (67). TAM receptors (Tyro3, Axl, and Mer) have been implicated in the innate immunity (68). Studies have demonstrated an obvious TAM shedding in DKD patients, though the mechanism of this observation remains unclear. Further research is warranted to establish the role of TAM in the development of renal injury and DKD.

## Angiotensin II

Angiotensin II (AngII) is also recognized as a mediator of hyperglycemia-induced renal damage. The concentration of renal Ang II is ~1,000-folds higher than that of circulating AngII (69). An increased AngII level is implicated in the development of renal fibrosis by directly upregulating the pro-fibrosis genes (70). Early studies have revealed that AngII induces cellular hypertrophy of tubular cells that is mediated by the activation of endogenous TGF-β (71, 72). Also, the study of primary PT has demonstrated that glucose significantly increases the concentration of AngII in cell lysates, while angiotensin receptor blocker (ARB) significantly reduces this effect of AgnII (73, 74). Furthermore, AngII induces ROS (75) and EMT (76, 77), leading to tubular cell damage. Importantly, recent studies have revealed a high affinity of angiotensin II type 2 receptor (AT2R) in the mitochondria of renal tubules. In the early stage of diabetes, AT2R inhibits the production of mitochondrial reactive oxygen species and cell proliferation. Overexpression of AT2R in tubular epithelial cells contributes to the decreased mitochondrial bioenergy efficiency and increased mitochondrial superoxide production (78).

In the current clinical practice, angiotensin-converting enzyme inhibitors (ACEI), and ARB are the first-line drugs being used in the prevention of DKD. Several recent studies have shown that the combination therapy of renin-angiotensin system (RAS) inhibitor together with neprilysin inhibitor was more effective in preventing renal fibrosis than using RAS inhibitor alone in the development of DKD [LCZ696 and angiotensin receptor blocker (79); combination of sacubitril [NEPi] and valsartan (80); combination of thiorphan [NEPi]/telmisartan [ARB]; and thiorphan/Dize [ACE2 activator] therapies (81)]. Moreover, the combination of PGE1 with ACE inhibitor protects renal function more as compared with PGE1 or ACEI monotherapy (82). These studies provide evidence on the alternative options of effective clinical treatment with RAS blockers.

## Fatty Acids

In healthy kidneys, ATP is primarily generated via oxidative phosphorylation (OXPHOS) of fatty acid (FA). However, in diabetics, the utilization of fatty acid is changed to glycolysis and lipid accumulation, which also represents an important pathway of DKD due to lipid accumulation in the renal tubular epithelia (83) via increased absorption and synthesis of fatty acids, in addition to decreased utilization. The toxic effect of FA on the renal tubular epithelial cells is associated with hypoxia and mitochondrial dysfunction (33, 84). A recent study has shown that FATP2, a member of the fatty acid transporter family, regulates DKD pathogenesis through a combined lipotoxicity and glucotoxicity (glucolipotoxicity) mechanism (85). Nevertheless, PBI-4050, which is a fatty acid receptor modulator, attenuates the development of DKD in type 2 diabetes (86). Saturated fatty acid (SFA)-related lipotoxicity is also the pathogenesis of diabetes-related PT cell damage. Therefore, increasing the enzymes that metabolize free fatty acid (FFA) can theoretically protect the PT cells from SFA-related lipotoxicity. The study by Iwai et al. has found a significantly lower expression of Stearoyl-CoA Desaturase-1 (SCD1) in the kidney of diabetic mice induced by a high-fat diet (HFD) than that of non-diabetic mice. Thus, enhancing SCD1-mediated desaturation of SFA and subsequent formation of neutral lipid droplets may provide a promising therapeutic target to reduce SFA-induced lipotoxicity (87). Besides, through restoring functional lymphatic vessels, SAR13175 was able to eliminate inflammatory cells and toxic lipid metabolites in the kidney that can also improve lipotoxicity-related fibrosis in diabetes (88).

## Autophagy

Autophagy is a highly conserved pathway through which cells degrade and recycle macromolecules and organelles. Growing evidence shows dysregulated autophagy in DKD (89). The well-known autophagy regulation pathways include mammalian target of rapamycin (mTOR), Adenosine 5′-monophosphate (AMP)-activated protein kinase (AMPK) and sirtuins (SIRT). In addition, a variety of stress conditions, including hypoxia, oxidative stress, ERS, and metabolism, have been shown to regulate autophagy (90, 91). In general, mild to moderate ERS and activation of autophagy play a protective role in kidney cells. When the harmful stimulus cannot be effectively alleviated, this leads to the sustained ERS creating an imbalance between ERS and autophagy. This will lead to kidney cell injury and progression of DKD (92).

mTOR can interact with several proteins to form two different complexes, namely mTORC1 and mTORC2, to regulate autophagy. There is ample evidence that mTORC1 is a key regulator of autophagy, which regulates different steps of autophagy such as nucleation, elongation, maturation, and termination (93). mTORC2 indirectly regulates autophagy by activating mTORC1. In general, mTORC1 is a negative regulator of autophagy by inhibiting the activity of Ulk1 complex through direct phosphorylation. On the contrary, AMPK and SIRT1 are effective positive regulator of autophagy (89). In recent years, some new findings have been made in this field. Huang et al. identified KCa3.1 (calcium-activated K+ channel) involved in renal tubular autophagy dysfunction through PI3K/Akt/mTOR signaling pathway in DKD (94). Theodomir et al. confirmed that P2Y2R deficiency increased the expression of sirtuin-1 and FOXO3a, which enhanced autophagy and improved renal insufficiency in DKD (95). In addition, Yang et al. found that Smad3, the downstream transcription factor activated by TGFβ (transforming growth factor β), suppressed lysosome biogenesis in a TFEB-dependent manner (96). Furthermore, ATF4 (activating transcription factor 4) (97), TRAIL (TNF related apoptosis inducing ligand) (98), Soluble epoxide hydrolase (sEH) and lys63 UB proteins were also confirmed to be involved in the regulation of autophagy in the kidney cells.

Targeting various components of autophagy pathway may become a new strategy for clinical treatment of DKD. As a potential target for regulating autophagy, Mikhail V blagosklony proposed rapamycin (sirolimus) for the treatment of diabetic kidney injury (99). However, clinical studies have found that rapamycin and its analogs can cause immunosuppression, glucose intolerance, increased risk of type 2 diabetes, and other side effects (100). In particular, it has been reported that long-term use of rapamycin can aggravate glomerular damage and increase albuminuria (101). Recently, Dudley W. Lamming group discovered the highly selective compound DL001, which inhibits mTORC1, could be developed for the treatment of DKD (100). In addition, several other drugs have been shown to improve DKD *in vivo* and *in vitro* models by regulating autophagy (102–104). SGLT2 inhibitors are also thought to increase autophagy in diabetic kidneys (105). The role of autophagy in the development of diabetes is still insufficient, and more experiments are needed to further elaborate in this field.

## Inflammation and EMT

In the development of tubulointerstitial fibrosis, the complicated process of inflammation not only is the initiating factor but also the result of the development of several other factors. Local inflammation in renal tubules is a marker of progressive renal disease (106). Additionally, systemic inflammation exists in patients with type 2 diabetes, which involves the production of a large variety of chemokines that promotes inflammation in the microenvironment, thus increasing renal damage. Inflammation promotes renal infiltration of monocytes and lymphocytes, which augments further the inflammatory response and the development of cell damage and fibrosis (71). Additionally, a large number of macrophages, lymphocytes, and mast cells infiltrate and secrete copious pro-inflammatory cytokines and oxygen-free radicals, which could provoke renal tissue damage and accelerate the process of renal fibrosis (107). Renal tubular inflammation is associated with several triggers, including local hyperglycemia, advanced glycation product, mitochondria oxidative stress, angiotensin II, PKC, and other factors (108). Recent evidence on the effects of histamines in renal function suggests that histamines may also contribute to glomerular hyperfiltration, inflammation, fibrosis, and tubule hypertrophy (109).

## Other Pathways Discovered in Recent Years

Numerous cell signaling pathways have been confirmed to play a role in the progress of DKD. Here, we discussed some of the new pathways discovered in recent years.

## Hippo Signal Pathway

The Hippo signal transduction pathway has been heavily researched in recent years. Experimental studies have shown the important roles of the Hippo signal transduction pathway in regulating organ size, carcinogenesis, tissue regeneration, and functions of stem cells. YAP (Yes-associated protein) and its homologous protein, TAZ (transcriptional coactivator with PDZ-binding motif), are the main effector molecules of the Hippo pathway. The study by Yang et al. has demonstrated that the activated YAP induced by the inhibition of MST1 up-regulates the activation of TEAD directly by binding to TEAD to form YAP-TEAD heterodimer, which promotes the expression of pro-fibrosis genes in the renal tubular epithelial cells (110). A high expression of YAP, TEAD, and CTGF was found in renal tissue of patients with type 2 DKD suggesting a key role of YAP in renal damage, while YAP expression is also correlated with Systolic BP, BUN, Cr, DKD stage, DKD pathological grade, serum albumin, and eGFR (111). The expression of YAP protein and its phosphorylation were also upregulated in the renal PTs of diabetic mice. Further studies have revealed that the activated EGFR-PI3K-Akt-CREB signaling pathway mediates the YAP gene expression, nuclear translocation, and interaction with the TEAD transcription factor complex (112). Besides, TAZ has been shown as a novel non-SMAD downstream effector of renal TGF-β1 signaling, which is activated in fibrotic kidney via TGF-β1-dependent mechanisms, while a sustained TAZ signaling promotes epithelial maladaptive repair (113).

## Nod-Like Receptors (NLRs)

NLRs are a family of cytoplasmic pattern-recognition receptors, which play several key roles in both innate and adaptive immunity (114–116) by inducing inflammation and cell death while facilitating rapid removal of invasive pathogens. Different NLRs poses distinct roles in regulating immunity and inflammation (117). NLRC3 inflammasome aggravates tubular injury through promoting pro-inflammatory and pro-fibrotic response of renal tubular cells (118). A study demonstrated that the reduction of NLRP3 inflammasome suppressed by the TNF-α inhibition alleviated tubular injury in DKD rats (119). Moreover, NLRP3 exerts inflammasome-independent effects on TGFβ signaling, which contributes to renal fibrosis in DKD (120). The role of NLRC5 has been shown to be multifaceted in the progression of DKD. Under high-glucose conditions, NLRC5 enhances IκB phosphorylation and reprograms macrophages toward the M1 phenotype in addition to activating the TGFβ signaling (121). Macrophages are closely related to interstitial fibrosis (122). Among the variety of phenotypes of macrophages, macrophages of M1 phenotype infiltrated the diabetic kidneys at the early stage play mainly the pro-inflammatory role, while the activation of macrophages M2 occurs in the late stage to promote renal fibrosis in DKD (123–125).

## PTEN

PTEN decreases in diabetic renal tubular epithelial cells when cultured with high glucose, contributing to impaired autophagy and renal fibrosis (126, 127). Animal studies have demonstrated that although the level of unmodified Pten decreases, the level of Pten^K27−polyUb^ increases significantly with the damaged renal tubules. Sufficient serine/threonine phosphatase activity can be obtained after the modification of Pten^K27−polyUb^ to remove the phosphate groups of TWIST, SNAI1, and YAP. Consequently, these pro-fibrosis transcriptional factors activate the pro-fibrosis genes (128). Li et al. have proposed that the unmodified PTEN (EMT prophylaxis) and Pten^K27−polyUb^ (EMT promotion) are dynamically regulated in kidney disease, in which the identification of Pten^K27−polyUb^ may help in the early diagnosis of DKD and represent a potential therapeutic target.

## Zinc Transporter

Zinc transporters are categorized into Zrt/Irt-related protein (ZIP) and zinc transporters (ZnT), which function together to maintain intracellular zinc homeostasis. In the cytoplasm, both ZIP and ZnT are zinc transfer proteins (129). Studies have demonstrated the subcellular localization of ZnT8 on the insulin secretory vesicle membrane of the islet β cells, which promotes the synthesis, storage, and secretion of insulin and regulates the homeostasis of intracellular free zinc ions. The study by Zhang et al. has found that ZnT8 is highly expressed in the tubular epithelial cells but only weakly expressed in the glomeruli or podocytes, and confirmed the protective effect of ZnT8 against tubulointerstitial fibrosis by inhibiting the TGF-β1/Smads signal pathway. However, in normal circumstances, overexpression, or knock-down of ZnT7 does not alter the phosphorylation level of Smad2/3 (130). On the other hand, Zhang et al. (52) suggest the important anti-fibrotic role of Zn via the PI3K/Akt/GSK-3β signaling pathway. Nevertheless, the role of Zn in the pathogenesis of DKD requires further research and clarification.

## Others

In addition to the above, several other signaling pathways have been studied in PT in DKD. These include FoxO1-STAT1 signaling (131), TSC1-mTORC1 signaling (132), HSP70-TLR4 axis (133), and PDGFRβ/Akt/mTORC1 nexus (134).

## MicroRNA

MicroRNA (miRNA) is a small molecule that attracts great interest in the field of DKD research, given that it has been implicated in the occurrence and development of DKD. In particular, miRNAs participate in the progress of tubulointerstitial fibrosis, leading to structural changes and dysfunction of renal tubules. Also, miRNA and RNA-induced silencing complex (RISC) form a complex (135), which inhibits the expression of target genes by promoting mRNA degradation or inhibiting mRNA translation. Thus, whether miRNA promotes or inhibits fibrosis will depend on their specific target genes related to fibrosis as summarized in the table below (Table 1).

**Table 1:** miRNAs related to tubulointerstitial fibrosis in diabetic kidney disease.

Oxidative stress	miR-25	PTEN (127) NOX4 (136)
	miR-146a	NOX4 (137)
	miR-4756	Sestrin2 (87)
Autophagy	miR-22	PTEN (127)
	miR-155-5p	Sirt1 (138)
EMT	miR-23a	SnoN (139)
	miR-27a	PARγ (140)
	miR-30b-5p	SNAI1 (141)
	miR-30c	SNAI1 (142)
	miR-30c-5p	JAK1 (143, 144)
	miR-34a-5p	SIRT1 (145)
	miR-98	Nedd4L (146)
	miR-130b	SNAI1 (147)
	miR-133b	SIRT1 (148)
	miR-145	ZEB2 (149)
	miR-181a-5p	Egr1 (150)
	miR-184	LPP3 (151)
	miR-192	Egr1 (152) ZEB1/ZEB2 (153)
	miR-199a-3p	IKKβ (154)
	miR-199b	SIRT1 (148)
	miR302a-3p	ZEB1 (155)
	miR let-7c	HMGA2 (156, 157)

## Biomarkers of Tubular Cell Injury

Several biomarkers of tubular cell injury have been identified in patients with DKD. TNFR1 and TNFR2 have been shown as reliable biomarkers for predicting the progression of DKD (158) and their levels also correlate with tubular cell injury and inflammation (159). Kim1 is known as an early biomarker for DKD and its level increases even prior to the onset of microalbuminuria (160). Urinary N-acetyl-beta-d-glucosaminidase (NAG) is also considered as a potential early biomarker for DKD (161).A cross-sectional study shows that u-NGAL and RBP-4 are potential markers of tubular damage which can be used as complementary measurements to albuminuria and GFR in the early diagnosis of DKD (162).

## Acute Kidney Injury (AKI) and DKD

Patients with DKD were susceptible to severe AKI and usually had a worse prognosis following AKI (163). Advani recently summarized clearly that diabetes may increase the risk of AKI while AKI may increase the risk of CKD in diabetes (164). PT suffers from more severe renal tubular hypoxia and mitochondria dysfunction in diabetic kidney. Inflammatory cytokines have been also reported to be upregulated in diabetic kidney leading to serial cascades of inflammation (165). Hyperglycemia, advanced glycation end products (AGEs) and albuminuria itself can induce the expression of adhesion molecules and chemokines in proximal tubular cells to aggravate injury. In a separate study (166), STZ-induced and Akita diabetic mouse models exhibited heightened susceptibility to increased tubule cell damage and programmed cell death caused by ischemia reperfusion injury (IRI). Proximal tubule cells exposed to high glucose exhibited higher apoptosis following depletion of ATP or exposure to severe hypoxia. The authors (166) identified activation of the intrinsic pathway of apoptosis characterized by mitochondrial Bax accumulation and cytochrome c release, and the activation of the intrinsic pathway of apoptosis which was induced by the upregulation of p53 in tubule cells exposed to high glucose and ischemic insult. The studies by Kelly et al. also showed that DKD patients are more susceptible to renal ischemia leading to more severe tubular cell apoptosis (167, 168).

In addition, miRNAs have been shown to be highly promising diagnostic markers for early DKD and it may have a potential role in the treatment of DKD. However, one miRNA often has multiple target genes, while one target gene may also be regulated by multiple miRNAs. Given this complexity, further studies are warranted to ascertain the specific roles of miRNA in renal fibrosis before considering their potentials application in clinical setting.

## Conclusions

**Figure 1:**
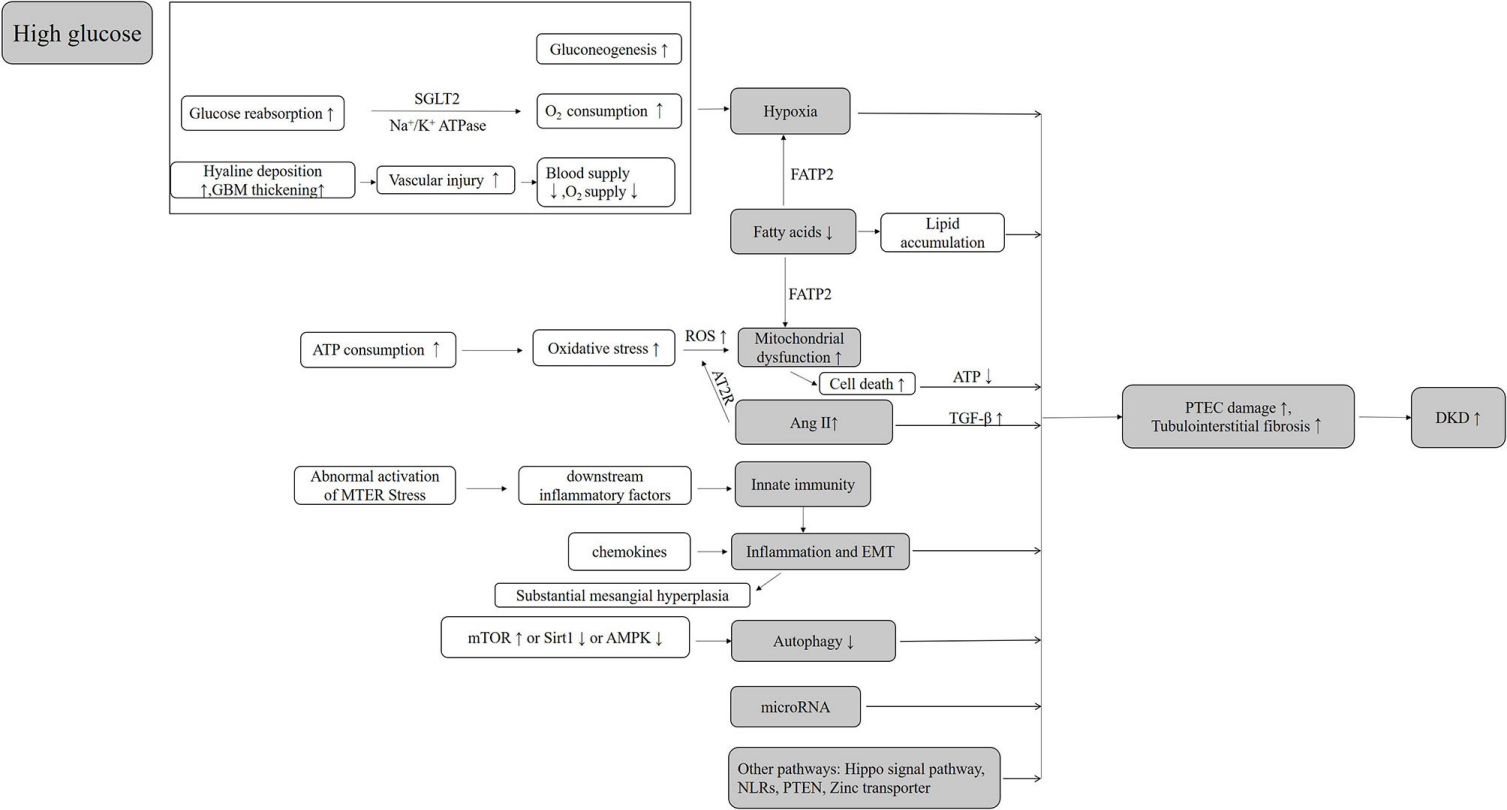
The mechanisms of tubular cell injury in DKD.

PT injury appears in the early stage of DKD and continues throughout the progression of DKD (169). Based on its structural and functional characteristics, PTs are vulnerable to injury in hyperglycemic states and difficult to recover. In diabetic patients, a high glucose transport state and local relative oxygen deficiency (primary and secondary) in PT may be the initial factors of tubular damage, while excessive mitochondria damages and ROS production are important contributors to the further damage of PTs in DKD. Abnormalities in hemodynamics, glucose and lipid metabolism, mitochondria, oxidative stress, inflammation, and many other factors interact with each other and form a vicious circle, leading to the renal tubular dysfunctions (Figure 1).

In this review, we discussed the potential mechanisms of renal tubular damage in DKD and potential therapeutic targets to prevent or treat the tubular cell injury. Renal tubular damage is a complex and dynamic process involving a “tubulocentric view” or “glomerulocentric view,” which represents a manifestation of different stages in the development of DKD. New studies are required to further understand the pathogenesis of tubular injury in DKD and to develop specific treatments to prevent and delay the tubular injury in DKD.

## Author Contributions

All authors listed have made a substantial, direct and intellectual contribution to the work, and approved it for publication.

## Conflict of Interest

The authors declare that the research was conducted in the absence of any commercial or financial relationships that could be construed as a potential conflict of interest.
